# Grover’s Disease Association with Cutaneous Keratinocyte Cancers: More than a Coincidence?

**DOI:** 10.3390/ijms25179713

**Published:** 2024-09-08

**Authors:** Roxana Nedelcu, Alexandra Dobre, Gabriela Turcu, Razvan Andrei, Elena Balasescu, Florentina Pantelimon, Mihaela David-Niculescu, Adina Dobritoiu, Raluca Radu, Georgiana Roxana Zaharia, Ionela Hulea, Alice Brinzea, Lorena Manea, Mihaela Gherghiceanu, Daniela Ion

**Affiliations:** 1 Pathophysiology Department, “Carol Davila” University of Medicine and Pharmacy, 050474 Bucharest, Romania; roxana.nedelcu@umfcd.ro (R.N.);; 2 Derma360 Clinic, 011273 Bucharest, Romania; florentina.pantelimon@gmail.com (F.P.);; 3 Oncologic Dermatology Department, Elias Emergency University Hospital, 011461 Bucharest, Romania; 4 Dermatology Department, Colentina Clinical Hospital, 020125 Bucharest, Romania; 5 Synevo, 014192 Bucharest, Romania; 6 Dermatology Department, “Grigore Alexandrescu” Emergency Pediatric Hospital, 011743 Bucharest, Romania; 7 Dermatology Department, Central Military Emergency Hospital “Dr.Carol Davila”, 010242 Bucharest, Romania; 8 National Institute for Infectious Diseases “Prof. Dr. Matei Balș”, 021105 Bucharest, Romania; 9 Dermatology Department, Pitié-Salpêtrière University Hospital, Public Assistance—Paris Hosiptals—AP-HP—Charles Foix, 75013 Paris, France; 10 Cellular, Molecular Biology & Histology Department, “Carol Davila” University of Medicine and Pharmacy, 050474 Bucharest, Romania; 11 Ultrastructural Pathology and Bioimaging Lab, Victor Babeş National Institute of Pathology, 050096 Bucharest, Romania

**Keywords:** cutaneous keratinocyte cancers, non-melanoma, basal cell carcinomas, cutaneous squamous cell carcinomas, Grover’s disease, transient acantholytic dermatosis

## Abstract

Better mechanistic understanding of desmosome disruption and acantholysis in Grover’s disease (GD) may improve management of this disease. Recent molecular studies highlighted promising pathways to be explored by directly comparing GD and selected features of associated skin diseases. The association between GD and cutaneous keratinocyte carcinomas, the most prevalent non-melanoma skin cancers (NMSC), is not completely characterized. To review the medical literature regarding GD-associated cutaneous keratinocyte cancers, focusing on molecular features, pathophysiological mechanisms, and disease associations, to help guide future research and patient management. GD has been associated with a variety of skin conditions, but its association with skin cancers has been rarely reported. Between 1983 and 2024, only nine scientific papers presented data supporting this association. Interestingly, we found that GD may mimic multiple NMSCs, as few authors reported GD cases misdiagnosed as multiple cutaneous squamous cell carcinomas for more than 4 years or the presence of superficial basal cell carcinoma-like areas associated with focal acantholysis. In conclusion: (a) GD may be an imitator of multiple NMSCs, and (b) the relationship between GD and NMSCs may reveal promising pathways for the mechanistic understanding of desmosome disruption and acantholysis in GD and may even lead to its reclassification as a distinctive syndrome.

## 1. Introduction

**Figure 1:**
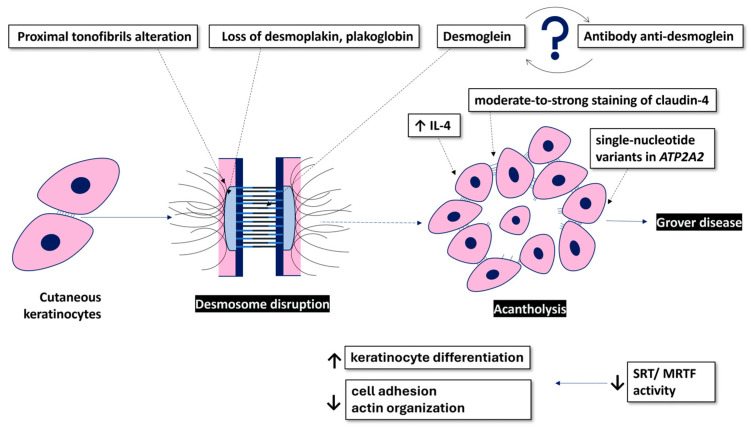
The molecular background of GD pathophysiology; ↓ decreased; ↑ increased.

Grover’s disease (GD), also known as transient acantholytic dermatosis, is an acquired papular dermatosis first mentioned in the medical literature in 1970 by Ralph Grover [1]. The accurate incidence and prevalence of GD are not available, but the true incidence is likely underestimated due to its similarity to several other conditions [2]. GD predominantly affects Caucasian middle-aged males, with a notable male-to-female disparity ranging from 3:1 to 7:1 [3]. Cases of Grover’s disease may be seen in Hispanic and black ethnic groups, but prevalence seems to be lower in darkly pigmented skin [4]. While diagnosis primarily relies on clinical presentation, histopathological examination serves as crucial support. The hallmark of GD is acantholysis (Figure 1), a phenomenon where intercellular connections such as desmosomes are lost and epidermal cells detach from one another, leading to characteristic lesions [5]. In normal skin, the intercellular space of desmosomes includes the glycoproteins desmogleins and desmocollins, which, in conjunction with the cytoplasmic counterparts plakoglobin and plakophilin, support intercellular attachment. Numerous diseases have been reported to be associated with GD, with many of them being oncologic in nature [6,7,8]. A study involving 72 patients with GD revealed concomitant skin cancer in 12% of subjects [7]. However, current evidence is insufficient to conclude a definitive correlation between skin cancers and GD.

A better mechanistic understanding of desmosome disruption and acantholysis would open the door for targeted molecular therapies and improved disease management. Recent molecular studies highlighted novel promising pathways to be explored, by directly comparing GD and selected features of associated skin diseases. However, the association between GD and cutaneous keratinocyte carcinomas, the most prevalent non-melanoma skin cancers (NMSC), is not completely characterized.

Here, we present insights into GD-associated keratinocyte carcinomas, focusing on molecular features, pathophysiological mechanisms, and the significance of the association for improved management of patients in the future.

## 2. Methods

To obtain a comprehensive view of the association between Grover’s disease (GD) and cutaneous keratinocyte carcinomas, we searched PubMed articles for the terms “Grover disease” OR “Grovers’s disease” OR “transient acantholytic dermatosis” AND “skin cancer” OR “keratinocyte carcinomas” OR “non-melanoma” OR “basal cell carcinoma” OR “squamous cell carcinoma” OR “actinic damage”. Between 1983 and 2023, eight articles were published on the topic (original studies, reviews, case reports, and case series), and, additionally, one press case report presented relevant data regarding this association.

## 3. Results

Table 1 summarizes core information within the 9 scientific papers that we found to contain data supporting the link between GD and NMSCs.

In a study group consisting of 72 patients with GD, the authors reported findings related to: hematoxylin-eosin-stained biopsy specimens; immunohistochemistry (IHC) for eight biopsy specimens stained for BRST-2, low-molecular-weight keratin CAM 5.2, and CD44; direct immunofluorescence; and indirect immunofluorescence used to detect major basic protein (MBP) [7]. Clinicopathologic correlations were discussed further, and the comorbidities summarized. In the study group, the authors identified all patterns of acantholysis recognized in the literature at that time (1999), with a predominance of pemphigus vulgaris-like patterns. A prevalent histological finding was the perivascular lymphocytic infiltrate, sometimes admixed with eosinophils, neutrophils, and plasma cells. Indirect immunofluorescence revealed large variation in eosinophil degranulation degree, staining one of the eosinophil granule mediators—MBP. Direct immunofluorescence detected changes considered non-specific, and IHC staining for markers of sweat glands was positive in 25% cases for CD44 and negative for BRST-2 and CAM 5.2. Of particular interest for our review is the consistent association between GD and cutaneous malignancies: 17% of patients with GD were diagnosed with concomitant skin cancer, and another 15% had concurrent dermatitis. Details about skin cancer subtypes or cells responsible for malignant proliferation at this level are not available from this study [7].

In another study, 33 cases of GD were analyzed with a focus on identification and interpretation of epidermal atypia present in GD cases. The research team described the presence of epithelial buds, nuclear pleomorphism, dyskeratosis, or an altered granular layer of the skin in histologically confirmed cases of GD [9]. Surprisingly, 64% of cases associated varying degrees of keratinocytic atypia, and the authors reported for the first time the presence of superficial basal cell carcinoma-like areas associated with focal acantholysis. The authors highlighted that dysmaturative pattern is not an uncommon phenomenon in GD and anticipated the challenge prominent squamous atypia may represent in order to differentiate between GD with epidermal dysmaturation pattern and cutaneous keratinocyte cancers or pre-malignant tumors such as actinic keratosis, Bowen’s disease, or basal cell carcinoma (BCC). The authors suggested that a useful clue for differentiating them is the confinement of the cleft to the basal layer in the acantholytic actinic keratosis in contrast with the cleft that affects all layers of epidermis in dysmaturative GD [9].

A few years later, a report of a 69-year-old man with a 4-year history of erythemato-papular rash on his trunk and extremities confirmed that some biopsies of GD could be mistaken for malignant epithelial lesions [8]. Multiple biopsies at the early stage of the eruption led to the diagnosis of multiple cutaneous squamous cell carcinomas (SCC), which was followed by appropriate surgical treatment. After these four years of disease, the authors reconsidered the case from multiple perspectives: the medical history of the patient and patient’s family were unremarkable; the eruption had a chronic course, worsening under exposure to heat and sweating; symptoms and systemic findings were absent; skin cancer screening was ambiguous; physical examination and dermoscopy revealed focally eroded papules and yellow crypts with white halos in a radiating pattern, all of these findings prompting a new biopsy. The main microscopic features included acanthosis, focal acantholytic dyskeratosis, parakeratosis and a perivascular lymphohistiocytic infiltrate. By correlating the medical history and clinical, dermoscopic, and microscopic characteristics of the case, extensive GD was diagnosed. The systemic and local treatment with retinoids and corticosteroids improved the disease course significantly [8].

Regarding the extensive or atypical form of GD, a systematic review presented morphological features and associations of disease. The extensive form of GD was characterized by the distribution of the eruption on the trunk plus at least one additional non-truncal area of the body. When analyzing 72 cases of extensive or atypical GD, the authors found it is much more frequently associated with a malignancy (in 61% of patients) compared with typical GD. Regarding the association of extensive GD with skin neoplasia, half of the nonhematologic cancers reported (12 cases) were metastatic melanoma [6]. Particularly interesting, even if NMSCs were not numerically reported in the review, two of the cited studies presented 10 male patients aged 38–63 years treated in various London and Paris hospitals between 1969 and 1993 with GD and multiple non-melanoma skin cancers (NMSCs) and/or actinic damage: BCCs, SCCs, actinic keratosis, and Bowen’s disease. The authors have proposed that the association between GD and multiple NMSCs would probably best be regarded as a distinctive syndrome [10,11].

In light of the above observations, we included in this review the first report of BCCs associated with GD in a middle-aged female (in press in Proceedings of EADV Congress 2024) [12]. A 62-year-old Caucasian female presented to our clinic with a disseminated eruption of pruritic papules that had developed and progressed continuously over the last 8 years. Initially diagnosed and treated as submammary intertrigo, the eruption subsequently slowly extended to involve the abdomen, lumbar region, and anterior and posterior thorax. During this time, some lesions self-resolved, but others appeared in crops, waxing and waning spontaneously for years. In the same year as the eruption onset, the patient observed an atypical lesion on the right flank, which was excised and diagnosed as superficial pigmented BCCs. Over the next few years, she developed seven additional BCCs at different locations: the first three were completely excised (on the forehead, right flank, and left calf), and four were treated with topical 5% imiquimod (on the lumbar region, right forearm, chest, and posterior thorax). All of the surgically excised BCCs were focally pigmented superficial subtypes. Histopathology showed: an apparently multifocal tumor proliferation in continuity with the overlying epidermis, represented by islands, nests, and anastomosing cords in the papillary dermis, composed of basaloid cells with an inverted nucleus-to-cytoplasm ratio and hyperchromatic nuclei with slightly irregular contours and relatively rare mitoses; palisading arrangement of tumor cells at the periphery; focal accumulation of melanin pigment within the cytoplasm; minimal desmoplastic reaction; maximum tumor thickness = 0.3 mm; peritumoral skin is slightly acanthotic, with areas of hyperortho- and parakeratosis, focal spongiosis, and moderate perivascular lymphomonocytic inflammatory infiltrate with rare interstitial eosinophils (eczematous aspect). The patient had no other personal history and no known family history of skin diseases or cancers. She reported occasional sun exposure during holidays.

During the physical examination, we observed Fitzpatrick type II skin phototype, multiple seborrheic keratoses, and a disseminated symmetrical eruption composed of erythematous papules with a whitish-scaling center, mainly on the trunk. Given the personal history of multiple BCCs and the peculiar clinical presentation with central hyperkeratotic papules, a biopsy was performed from an abdominal papule. Histopathological examination revealed: hyperparakeratosis and moderate acanthosis with unevenly elongated epidermal ridges; dyskeratosis with the formation of round and granular bodies; foci of acantholysis with intraepidermal cleavage; moderate lymphocytic inflammatory infiltrate, including eosinophils, vaguely distributed perivascularly and diffusely interstitially in the upper dermis, consistent with GD, displaying Darier-like characteristics. Dermoscopic evaluation revealed an erythematous papule with a central stellar-like, yellowish keratotic plug. Based on the evolution (chronic course), clinical, dermoscopic, and histopathological exams, and the negative family history of hereditary diseases, GD with a Darier-like pattern was diagnosed.

To our knowledge, there are no reported cases documenting the coexistence of multiple BCCs with GD in a middle-aged female. Moreover, the predominance of GD in males makes this case exceptional. The clinical diagnosis was particularly challenging due to the atypical presentation of the papules, which diverged from the classic descriptions of GD, as keratotic lesions are rarely mentioned in the literature [4]. The difficulty in distinguishing GD from NMSCs, especially on the trunk, emphasizes the importance of comprehensive evaluation, including histopathology, to guide appropriate management. Given the well-established association between BCCs and immunosuppression [13] and considering some case reports of GD associated with non-skin cancers, our patient necessitated careful assessment to rule out any underlying malignancies.

Two other recently published articles addressed the coexistence of GD and bullous diseases in patients with aggressive cutaneous carcinomas [14,15]. Pinto-Pulido et al. reported the case of a 90-year-old man with advanced earlobe SCC treated with a PD-1 inhibitor who developed a pruritic eroded eruption on the neck and abdomen after two months from therapy initiation, with the extension of the lesions on the back and legs during the next month. The coexistence of GD and bullous pemphigoid (BP) was supported by histologic and direct immunofluorescence (DIF) features. Histology revealed parakeratotic hyperkeratosis, suprabasal acantholysis, and dyskeratosis, while DIF showed strong linear C3 and IgG positivity along the basement membrane [14]. Another recent article reported association of GD with pemphigus foliaceus observed in the case of a 63-year-old woman with a recurrent BCC on the right lower eyelid with infiltration of the orbital floor and compromise of the rectus and inferior oblique muscles, treated with an antagonist of the smoothened receptor (SMO) that inhibits the Hedgehog signaling pathway and extensive surgery. A few months after the treatment, the patient developed an extensive erythemato-squamous eruption involving surgery scars, trunk and face. Multiple skin biopsies revealed superficial blisters with acantholytic cells, “corps ronds”, and grain formation, dyskeratosis. DIF showed an intercellular IgG deposition. By correlating the microscopic and molecular results with the clinical course of the disease, the authors considered the eruption to be the expression of concurrent GD and pemphigus foliaceus [14].

**Table 1:** Summary of studies investigating Grover’s disease (GD) and cutaneous malignancies.

Scheme	Study Type	Sample Size	Core Findings	Ref.
Davis et al. (1999)	Original article	72	A total of 12 of patients with GD (17%) had an associated skin cancer but details about the skin cancer subtype were not available from this study	[7]
Aljarbou et al. (2018)	Original article	33	A total of 20 cases (64%) of GD biopsies associated varying degrees of keratinocytic atypia that may represent a challenge to differentiate between GD with dysmaturative pattern and cutaneous keratinocyte cancers or pre-malignant tumors such as actinic keratosis (AKs), Bowen’s disease, or basal cell carcinoma (BCC).	[9]
Kotzerke et al. (2021)	Case report	1	A 69-year-old man misdiagnosed with multiple squamous cell carcinomas (SCCs), later identified as extensive GD after multiple biopsies and comprehensive analysis.	[8]
Gantz et al. (2017)	Systematic review	72	Extensive or atypical GD is associated with malignancy in 31 of cases (61%); half of non-hematologic cancers were metastatic melanoma (six cases).	[6]
Fawcett HA and Miller JA (1983)	Case series	10	A total of 9 of 10 (90%) male patients were associated with at least one of the following: BCCs (six patients), AKs (four patients), SCCs including Bowen’s disease (two patients), and melanoma (one patient).	[10]
Mokni M et al. (1993)	Case report	1	A 58-year-old man diagnosed with persistent acantholytic dermatosis associated with multiple AKs, SCCs, BCCs, and Bowen’s disease.	[11]
Dobre et al. (2024)	Case report	1	A 62-year-old female with Darier-like pattern GD coexisted with eight BCCs.	[12]
Pinto-Pulido et al. (2023)	Case report	1	A 90-year-old male developed GD and bullous pemphigoid after 2 months of PD-1 inhibitor treatment for invasive SCC.	[14]
Magdaleno-Tapial et al. (2019)	Case report	1	Coexistence of GD with pemphigus foliaceus after invasive relapsing BCC treatment.	[15]

## 4. Discussion

### 4.1. Grover’s Disease (GD)—From Clinical to Molecular Understanding

The prototypical presentation of GD manifests as a sudden onset of self-limited, pruritic papular or papulo-vesicular eruptions primarily localized on the trunk followed by extremities [3]. Other areas involved less frequently include the neck (21%), face/scalp (17%), axilla (4%), and mucosal membranes (1%). When the rash spreads to additional body parts beyond the trunk, it is categorized as extensive GD, while involvement of at least one non-truncal area characterizes atypical GD [6]. Despite its historical designation as “transient acantholytic dermatosis”, it encompasses three main clinical forms: transient eruptive, persistent pruritic, and chronic asymptomatic [2]. The duration has been correlated with age, as older individuals are more likely to have extensive, longer-lasting eruptions. “Grover’s disease” is the more commonly used term and may be more appropriate since the disease has demonstrated persistence and various morphologies beyond acantholysis [16].

According to existing studies, dermoscopy has emerged as a valuable tool in aiding the diagnosis of GD and also providing insights into disease subtypes [17]. Typically, dermoscopic examination reveals centrally tan to brown keratotic areas with a star-like or discrete roundish/linear appearance, often surrounded by a whitish halo against a background ranging from white to pink to tan [17,18,19]. It was shown that the Darier-like subtype corresponds with this pattern, whereas the spongiotic subtype is characterized by a yellowish-red background and white scales [17].

The characteristic histologic features of the disease include focal acantholysis and varying degrees of dyskeratosis [20]. The histology of GD can present challenges in diagnosis due to predominantly focal involvement and its varied appearances, displaying various proportions of different intraepidermal acantholytic patterns [21]. In addition to classical histopathological subtypes such as Darier-like, spongiotic, Hailey–Hailey-like, and pemphigus vulgaris/foliaceus-like [20], recent descriptions have included subtypes like dysmaturative, porokeratotic, vesicular, lichenoid, and pseudoherpetic, which may manifest in isolation or combination [2,22]. Dermal infiltrate typically consists of lymphocytes and eosinophils. Establishing a definitive diagnosis may require analyzing multiple biopsy specimens, as they often exhibit non-specific features or closely resemble conditions such as folliculitis, actinic keratosis, drug eruptions, or insect bites [2].

From a transmission electron microscopy perspective, the tonofibrils in GD are poorly developed, with damage seeming to initiate proximal to their insertion in desmosomes [23]. While the structure of desmosomes in the spinous layer appears normal, their numbers vary across studies, ranging from decreased [24] to increased numbers [25]. The latter indicates a potential compensatory mechanism for defective desmosomal function [25]. Notably, half-split desmosomes, observed in diseases like pemphigus vulgaris/foliaceus or Darier’s disease, are absent in GD [25,26]. A plausible explanation for acantholysis could be the impairment of desmosomal plaque proteins linking tonofilaments to desmosomal cadherins [25].

### 4.2. The Intriguing Puzzle of Grover’s Disease Pathophysiology

Despite its initial mention many years ago and the well-deciphered clinical features since then, the precise etiology and pathophysiology of GD remain unclear. Some triggers, such as sweating, heat, sunlight, ionizing radiation, and prolonged bed rest, have been noted in many cases [3,5]. Over time, numerous hypotheses have emerged regarding its pathogenesis, but some have been contradicted by subsequent studies, underscoring the ongoing challenges in understanding this enigmatic dermatological disorder (Figure 1).

One of the earliest pathogenic theories proposed for GD suggested that occlusion of damaged intraepidermal eccrine ducts led to the characteristic skin eruption. It was hypothesized that this occlusion caused leakage of molecules into the epidermis, triggering acantholysis. However, subsequent studies contradicted this hypothesis: (1) no histological/IHC evidence of a relationship between the acantholytic areas and the eccrine apparatus were identified—no leakage of sweat molecules, such as carcinoembryonic antigen or epithelial mucins, have been demonstrated [7,21,27,28]; (2) GD tends to spare the acral surfaces, where a high number of eccrine glands exist [21]. 

Other authors suggest that GD may arise from alterations in skin integrity rather than direct epidermal destruction, such as significant xerosis [13,16]. Recently, Bui et al. demonstrated moderate-to-strong staining of claudin-4 in disrupted keratinocytes both surrounding and within the acantholytic and bullous areas in acantholytic disorders, including cases of GD [29]. In non-affected areas, keratinocytes located in the stratum granulosum, as well as those surrounding hair follicles and adnexal glands, exhibited a normal staining pattern characterized by weak-to-moderate expression in all cases included in this study. The distinctive staining pattern described by them displayed a tapering effect as it transitioned away from the disrupted regions [29]. Tight junctions (TJs), which are predominantly located in the granular cell layer of skin, are essential for maintaining the epidermal barrier, ensuring intercellular integrity, and regulating paracellular permeability. In addition to their barrier function, TJs are involved in keratinocyte proliferation, differentiation, adhesion, and apoptosis [30]. Claudins, a diverse family of integral membrane proteins, are critical components of TJs and exhibit specific expression patterns across different cells and tissues, which are crucial for the selective permeability of each tissue. In particular, claudin-1 and claudin-4, which show a distinct expression pattern in the stratum granulosum, regulated by the ∆Np63 transcription factor from the p53 family, are vital for skin barrier functions [31,32]. The knockdown of claudin-4 in mice results in severe transepidermal water loss and early death, underscoring its crucial role in maintaining an effective epidermal barrier. Moreover, Li et al. demonstrated that the significant upregulation of claudin-4 could act as a compensatory mechanism to maintain barrier function in affected skin regions, potentially counteracting the barrier disruption caused by the downregulation of claudin-1 in these disorders [33].

In the case of linear GD, there is a hypothesis suggesting that the disease’s cause might reveal a previously hidden tolerance to abnormal clones, often keratinocytes, nestled within specific embryonic Blaschko lines [25]. Furthermore, a study demonstrating the loss of desmoplakin and plakoglobin in desmosomes in GD has led to a hypothesis that this disease may result from an impairment of the dynamics of desmoplakin or other desmosomal plaque proteins [25,34]. 

Some studies have proposed an immunomediated pathogenesis of GD [5,35]. However, it remains unclear whether the reported antibodies against desmogleins are the cause of GD or if they appear as a consequence of GD, representing an immune response to the disintegrated desmosomes due to acantholysis [35]. The most recent studies have revealed intriguing findings regarding GD. The transcriptomic profile of GD, Hailey–Hailey disease, and Darier disease exhibits a significant overlap, indicating an increase in keratinocyte differentiation and a decrease in cell adhesion and actin organization [36]. The downregulation of the latter was suggested to occur as a result of decreased serum response factor/myocardin-related transcription factor (SRF/MRTF) activity. Importantly, these characteristics were not observed in other inflammatory disorders such as psoriasis and atopic dermatitis [36].

Because Grover’s disease has been reported after checkpoint (cytotoxic T-lymphocyte-associated protein 4-CTLA4) inhibitor therapies, and patients receiving these therapies have been found to have higher serum levels of interleukin-4 (IL-4), it was hypothesized that the upregulation of this interleukin plays an important role in the pathogenesis of GD [36]. This theory is further supported by the successful treatment of refractory cases of Grover’s disease with dupilumab [37,38,39]. Dupilumab is a human monoclonal IgG4 antibody that targets and inhibits IL-4 and interleukin-13 (IL-13) signaling by specifically binding to the IL-4Rα subunit common to both IL-4 and IL-13 receptor complexes [40]. The positive response to this therapy indicates that type 2 inflammation may be involved in the pathogenesis of GD. Given that age-related changes in the immune system can lead to a pro-inflammatory state with dysregulation of the Th1/Th2 balance towards a Th2-inflammation profile, this type 2 inflammation may also help explain the increased incidence of GD with age [38].

The associations of GD with other coexisting dermatoses and medications are still being investigated to better decipher their possible etiopathogenic involvement in the course of GD. In addition to the immune checkpoint inhibitors, other medications have been reported in association with GD, such as cetuximab, anastrazole, BRAF inhibitors, and antimicrobial therapy [41,42].

Another recent study, which performed genetic analysis on 15 archival tissues from patients with GD, found that 80% were associated with somatic damaging single-nucleotide variants (SNVs) in *ATP2A2*. Notably, UV light-induced mutagenesis may have contributed to the development of the lesions because all variants were C > T or G > A substitutions. This finding underlines the role of somatic mutations in acquired diseases [43]. SERCA2, a sarco/endoplasmic reticulum Ca^2+^-adenosine triphosphate (ATP)ase pump encoded by the *ATP2A2* gene, plays a crucial role in maintaining homeostatic cytoplasmic Ca^2+^ levels. Defects in *ATP2A2* associated with Darier disease result in both acantholysis and apoptosis, which are partly due to alteration in the synthesis, trafficking, and folding of desmosomal proteins, as well as abnormal cytokeratin expression [44]. Unlike GD, Darier disease is an autosomal dominant condition that manifests earlier in life. Additionally, it has been hypothesized that some cases of GD might actually be mosaic forms of Darier disease [43].

To date, there have been no mechanistic studies of GD, nor have there been studies utilizing GD patient-derived keratinocytes, resulting in limited understanding of desmosomal dysfunction in this disease. Furthermore, this knowledge gap is compounded by the absence of an animal model closely resembling the symptomatology of the human disease [36].

### 4.3. Grover’s Disease (GD) Association with Cutaneous Keratinocyte Carcinomas

The association between GD and NMSCs is far from being completely elucidated, but there is sufficient scientific evidence to support the relevance of continuing studies on this topic.

On the one hand, there are several authors who have emphasized the challenge to differentiate between GD with keratinocyte atypia in the early stages and NMSCs. In this regard, Aljarbou and colleagues examined biopsy specimens from GD cases, focusing on atypical histopathological changes, revealing in over 60% of cases actinic keratosis-like changes, nuclear pleomorphism, dyskeratosis in all layers of epidermis, and an altered granular layer [9]. A few years later, Kotzerke and team confirmed that GD could be a multiple NMSCs mimicker: the authors reconsidered the diagnosis of multiple cutaneous SCCs after 4 years of monitoring the case of a 69-year-old man who had multiple biopsies [8]. Davis et al. reported in a retrospective study conducted at Mayo Clinic Rochester that 12 (16.6%) of 72 cases of GD had skin cancer as a concurrent condition [7]. Details about the clinical or microscopic features of the 12 cutaneous malignancies associated with GD were missing and would have been valuable, as the above authors raised awareness recently regarding the challenge to differentiate between GD with epidermal atypia and cutaneous keratinocyte cancer [8,9].

On the other hand, several papers highlighted the link between actinic damage, the medical history of NMSCs, and GD (Table 1). We elected to include our own case of multiple BCCs associated with GD (in press to EADV Congress 2024) [12]. This case is of a 62-year-old female who developed eight basal cell carcinomas and persistent GD during an almost ten-year period. To our knowledge, this is the first association between Darier-type GD and multiple BCCs in a female patient with no significant condition in her past medical history. Few authors have previously described the coexistence of GD and multiple BCCs in a case series of male patients [10,11]. A number of authors presented the coexistence of GD and single cutaneous carcinoma, rather associated with different potential etiopathogenic factors of GD [14,15]. Notable from the literature is the case of a 63-year-old woman with a recurrent BCC on her right lower eyelid, treated with vismodegib and large exenteration. Three months after surgery, the patient was diagnosed with concurrent GD and pemphigus foliaceus sustained by clinical, histological, and immunofluorescence examinations [15]. Another case report presented a 90-year-old man treated with pembrolizumab for advanced earlobe SCC who simultaneously developed GD and bullous pemphigoid two months after initiating pembrolizumab [14].

## 5. Conclusions

We reviewed the medical literature regarding GD-associated cutaneous keratinocyte cancers, focusing on molecular features, pathophysiological mechanisms, and disease associations, to help guide future research and patient management. GD has been associated with a variety of skin conditions, but its association with skin cancers has been rarely reported. Between 1983 and 2024, only eight articles presented data supporting this association. 

Two key points emerged from reviewing the medical literature regarding GD-associated keratinocyte carcinomas: GD may be an imitator of multiple NMSCs; the accurate association between GD and NMSCs may reveal promising pathways for mechanistic understanding of desmosome disruption and acantholysis in GD and may be regarded as a distinctive syndrome. We suggest that the newly identified association between GD and multiple NMSCs may offer new insights through further comprehensive and comparative molecular studies of GD with epidermal atypia and cutaneous carcinogenesis. Our analysis also represents a meaningful support for clinicians to consider the differential diagnosis of GD in patients with multiple NMSCs when no risk factor is identified and a pruritic, erythematous papular, or papulovesicular rash is observed at any time during the patient monitoring.
